# Physicians' needs for drug allergy documentation in electronic health records and allergy alert systems: Results of an end user's survey

**DOI:** 10.1002/clt2.12141

**Published:** 2022-04-06

**Authors:** Katoo M. Muylle, Sven Van Laere, Martine Grosber, Pieter Cornu

**Affiliations:** ^1^ Department of Pharmaceutical and Pharmacological Sciences (FARM) Vrije Universiteit Brussel Research Group Clinical Pharmacology & Clinical Pharmacy (KFAR) Brussels Belgium; ^2^ Department of Public Health (GEWE) Vrije Universiteit Brussel Research Group of Biostatistics and Medical Informatics (BISI) Brussels Belgium; ^3^ Department of Gerontology (GERO) Vrije Universiteit Brussel Research Group of Skin Immunology and Immune Tolerance (SKIN) Brussels Belgium; ^4^ Department of Dermatology Universitair Ziekenhuis Brussel Brussels Belgium; ^5^ Department of Medical Informatics Universitair Ziekenhuis Brussel Brussels Belgium

**Keywords:** allergy documentation, allergy alert systems, drug allergy, electronic health records

To the Editor,

Drug allergies are perceived as an important public health problem.^1^ Clinical decision support systems (CDSS) for drug allergy screening have the potential to prevent administration of a drug to a patient with a documented allergy or hypersensitivity to that or a similar drug, thereby preventing possible adverse drug events.^2^, ^3^ However, mainly because of the poor quality and accessibility of the source documentation, specificity of drug allergy alerts is low and alert override rates often exceed 90%.^3^ In our institution, a general university teaching hospital with a self‐developed electronic health record (EHR; see Figure 1), allergens can be recorded through free‐text or through selection from a limited selection list with optional recording of a date and severity level. Currently, there is no CDSS for allergy screening, but physicians are familiar with the concept of receiving safety alerts at prescription as a CDSS for drug‐drug interaction screening is already used.

**FIGURE 1:**
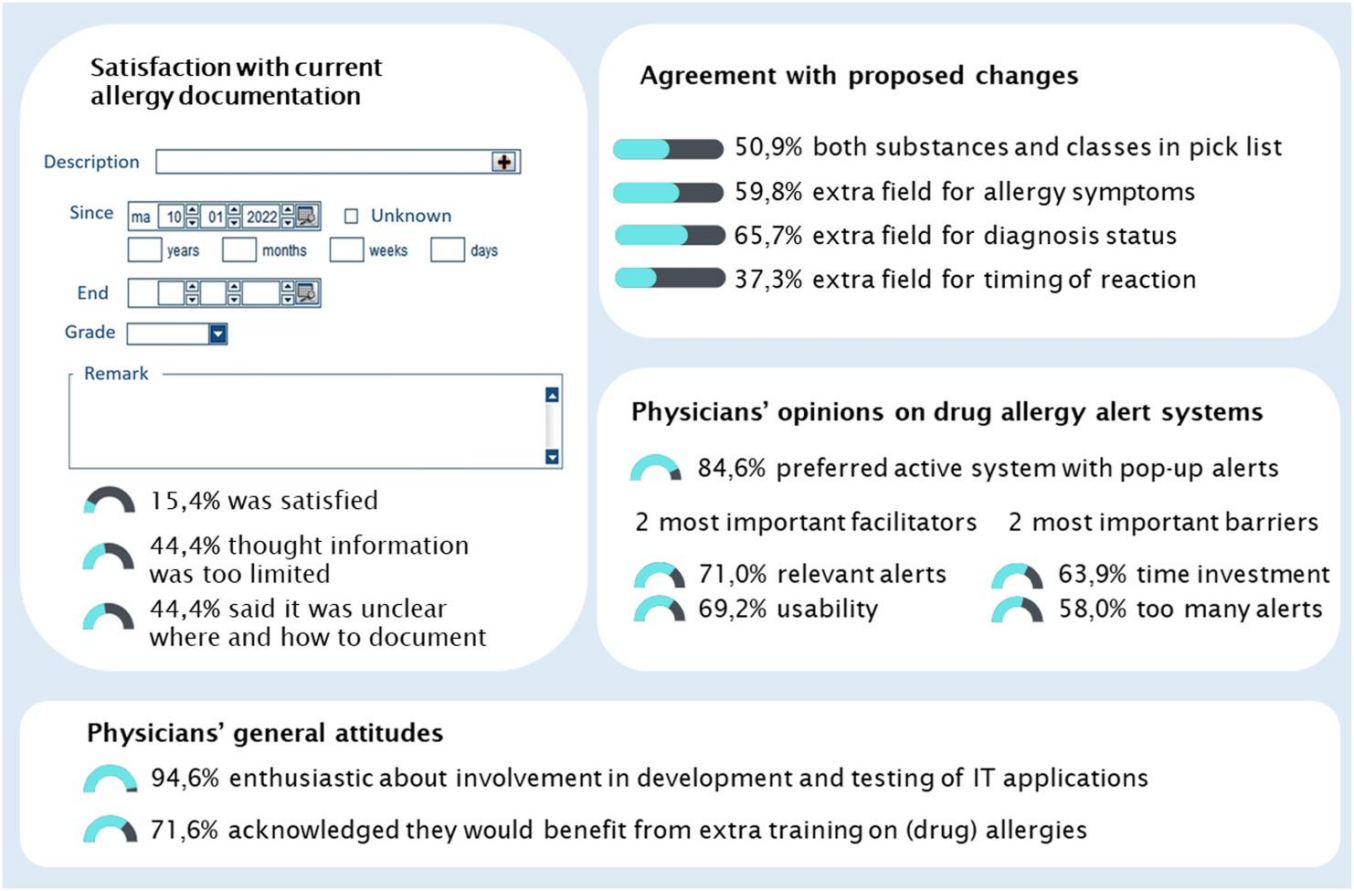
Current implementation of allergy documentation module and overview of main survey results

Before implementing a CDSS for drug allergy screening, we performed a survey about physicians’ attitudes and expectations towards allergy documentation and drug allergy alert systems. The survey was used to identify problems with the current allergy documentation and opportunities for improvement. The survey study was approved by the ethical committee of the University Hospital Brussels with reference number BUN143202043018.

## 1 SURVEY RESULTS (FIGURE 1)

Of 501 contacted prescribing physicians, 169 (33.7%) completed the electronic questionnaire anonymously on the LimeSurvey platform. Ninety Seven (57.4%) of respondents were female and 128 (75.7%) were physicians from non‐surgical departments (e.g. emergency department, pediatrics, internal medicine). Experience levels were evenly distributed: 49 (29.0%) respondents had <5 years of experience, 34 (20.1%) respondents 5–10 years, 44 (26.0%) respondents 11–20 years and 42 (24.9%) respondents had >20 years of experience.

Only 26 (15.4%) respondents were satisfied with the current allergy documentation module. Seventy‐five (44.4%) respondents thought that the current allergy documentation was insufficient and that it was unclear how and where to document an allergy in the EHR. Several respondents additionally commented in free‐text that allergy information is often wrong and incomplete and that the information is hard to find. Currently, only drug classes (e.g. penicillin) can be selected, but 86 (50.9%) respondents preferred having both substances and drug classes in the selection list. Furthermore, 101 (59.8%) respondents wanted a separate field to document allergy symptoms and 111 (65.7%) a field to indicate whether the drug allergy was confirmed by a physician and/or a diagnostic test. Only 63 (37.3%) respondents wished a separate field to indicate whether it was an immediate or delayed type reaction.

Concerning CDSS, 143 (84.6%) physicians preferred an active system (i.e. pop‐up allergy alerts) over a passive system where the prescriber needs to click on a specific icon to access advice. The most important barriers for using CDSS were time investment to handle alerts (108%, 63.9%) and receiving too many alerts (98%, 58.0%). Clinically relevant alerts (120%, 71.0%) and usability (117%, 69.23%) were the two most important facilitators for CDSS for drug allergy screening.

The vast majority (160%, 94.7%) agreed that involving physicians in development and testing of information technology (IT) applications improves the usability and correct use of such applications in clinical practice. Several respondents spontaneously volunteered for further involvement in redesigning the allergy documentation. Most respondents in our institution are open to educational offers since 121 (71.6%) indicated they would benefit from extra training on (drug) allergies.

## 2 DISCUSSION

Physicians were dissatisfied by the current documentation of drug allergies in our EHR resulting in fragmented, incomplete and inaccurate allergy documentation. This is in line with reports from other institutions.^3^, ^4^, ^5^, ^6^ It is crucial to standardize allergy documentation in structured data entries in EHRs avoiding free‐text entries as much as possible, not only to improve the quality of the information, but also to improve sharing of allergy information across different institutions.^3^, ^4^ There is still no general consensus among allergy experts on what exactly to document, but a more detailed specification including the allergen, a clinical description of the reaction, an (approximate) event date and a status indicating whether the association is confirmed or suspected seems agreed upon.^4^, ^6^, ^7^ There is a preference to document substances rather than drug classes.^4^ Next to the content, the development of a good graphical user interface is crucial for successful translation into clinical practice. Luri et al. found that 93% of the currently used drug allergy alerts systems were active, that is, pop‐up allergy alerts interrupted the workflow.^2^ Physicians in our hospital also preferred such an active system. Efforts to increase CDSS’ efficiency should be focused on more clinically relevant alerts and a better usability.^2^, ^3^, ^4^ Education of healthcare professionals and patient engagement is another important strategy to improve the accuracy of allergy entries and to enhance allergy delabeling practices.^4^, ^6^, ^8^ About 70% of respondents indicated that there was a need for additional training on drug allergies.

## 3 CONCLUSION

Physicians were dissatisfied with the quality of the current allergy documentation and appreciated being asked for feedback and ideas for improvement. Involving physicians in the development and testing of new IT applications could increase the acceptance and willingness to use these applications in clinical practice. The most important facilitators for CDSS for drug allergy screening were clinically relevant alerts and usability. Results of this survey will be used to redesign the allergy documentation module with improved quality and usability as the basis for a CDSS with clinically relevant alerts.

## CONFLICT OF INTEREST

The authors declare they have no conflict of interest.

## AUTHOR CONTRIBUTIONS

**Katoo M. Muylle:** Conceptualization; Equal, Data curation; Lead, Formal analysis; Lead, Funding acquisition; Equal, Investigation; Equal, Methodology; Equal, Project administration; Lead, Visualization; Lead, Writing – original draft; Lead, Writing – review & editing; Equal. **Sven Van Laere:** Conceptualization; Equal, Data curation; Supporting, Investigation; Equal, Methodology; Equal, Visualization; Supporting, Writing – review & editing; Equal. **Martine Grosber:** Conceptualization; Equal, Investigation; Equal, Methodology; Equal, Visualization; Supporting, Writing – review & editing; Equal. **Pieter Cornu:** Conceptualization; Equal, Funding acquisition; Equal, Investigation; Equal, Methodology; Equal, Project administration; Supporting, Supervision; Lead, Visualization; Supporting, Writing – review & editing; Equal.

## Data Availability

Data available on request from the authors.
